# Water Management for *μ*DMFC with Foamed Stainless Steel Cathode Current Collector

**DOI:** 10.3390/nano12060948

**Published:** 2022-03-14

**Authors:** Fan Zhang, Yanhui Zhang, Zhengang Zhao

**Affiliations:** 1 Faculty of Information Engineering and Automation, Kunming University of Science and Technology, Kunming 650500, China; zhangfan525025@163.com (F.Z.); yanhui_zhang01@163.com (Y.Z.); 2 Yunnan Key Laboratory of Green Energy, Electric Power Measurement Digitalization, Control and Protection, Kunming 650000, China

**Keywords:** micro direct methanol fuel cell, water management, cathode current collector, foamed stainless steel, gradient wettability

## Abstract

For micro direct methanol fuel cell (*μ*DMFC), water flooding on the cathode seriously affects the performance stability. Additionally, the effect of material and wettability of the cathode current collector (CCC) on the drainage capacity is studied to improve the *μ*DMFC’s performance. To this end, a CCC with foamed stainless steel was prepared to assemble the *μ*DMFC due to its absorbency. Further, based on analyzing the gas–liquid two-phase flow characteristics of the *μ*DMFC cathode, it was found that the gradient wettability CCC could accelerate the discharge of cathode water. Hence, the foam stainless steel CCC was partially immersed in a KOH solution to complete the gradient corrosion using its capillary force. Then, four different types of gradient wettability CCC were prepared by controlling the time of chemical corrosion. Finally, the performance of the *μ*DMFC with different gradient wettability CCC was tested at room temperature using electrochemical impedance spectroscopy (EIS) and discharge voltage. The experimental results show that the gradient wettability CCC can improve the performance of the *μ*DMFC by slowing down the rate of cathode flooding. The optimum corrosion time is 5 min at a concentration of 1 mol/L. Under these conditions, the CCC has the best gradient wettability, and the *μ*DMFC has the lowest total impedance. The discharge voltage of the *μ*DMFC with corroded CCC is increased by 33.33% compared to the uncorroded CCC *μ*DMFC. The gradient wettability CCC designed in this study is economical, convenient, and practical for water management of the *μ*DMFC.

## 1. Introduction

With the rapid development of industry, the rate of consumption of fossil fuels has been dramatic. The micro direct methanol fuel cell (*μ*DMFC), which relies on an internal redox reaction to generate electricity, provides a green route for environmental and energy concerns [1,2,3]. To effectively improve the performance of the *μ*DMFC, in-depth research into cathode flooding should be implemented because excess water can severely block the gas channels and prevent O_2_ from reaching the reaction sites uniformly [4,5,6,7]. Therefore, water management of the *μ*DMFC is still a key technology [8,9,10,11].

To analyze the emergence of water flooding and the hazards it brings, scholars have developed different physical models for the gas–liquid two-phase flow in the cathode of fuel cells [12,13,14,15,16]. In 2020, Mehnatkesh et al. [17] used a deep neural network model to measure water coverage in fuel cells. The distribution of water in the flow field and the identification of areas of water accumulation in the gas channels were analyzed. In 2021, Rubio et al. [18] proposed a fuzzy model to determine the extent of fuel cells flooding or dehydration in real time. Salaberri et al. [19] used a pore grid model to analyze the relative effect of local water blockage on the gas diffusion layer and convection. Building the physical model of the gas–liquid two-phase flow in the fuel cell cathode is an important research direction [20,21]. However, experimental investigations are an essential part of scientific research, and other scholars have directly observed the water flooding phenomenon at the fuel cell cathode through experiments [22,23,24]. In 2020, Rahimi et al. [25] designed a transparent fuel cell stack and studied the effect of water flow state on voltage and pressure by visualization. In 2021, Chasen et al. [26] combined X-ray imaging and EIS to measure the water in the fuel cell flow field, which showed that the water content of the parallel flow field was much higher than that of the serpentine flow field. Based on theory and experimental investigations, scholars have designed different structures in the membrane electrode and cathode flow fields of fuel cells to mitigate water flooding [27,28]. In 2018, Fly et al. [29] analyzed the fluid distribution in the flow field of the metal foam fuel cell. The experimental results showed that the velocity of water covering the flow field was enhanced by 61% using the foam flow field. In 2019, Yuan et al. [30] used carbon aerogel to construct a water management layer in the membrane electrode of the fuel cell, and they found that the layer enhanced water recovery. In 2020, Karthikkyan et al. [31] placed porous inserts in the flow field to alleviate cathode flooding and showed that the insertion methods and insert specifications had significant effects on the performance of the fuel cell. Sun et al. [32] used foam metal instead of conventional current collectors and found that modifying the foam metal current collector with wettability could improve the performance of the fuel cell. Overall, the application of treated porous metals to *μ*DMFC can effectively mitigate cathode flooding.

In this study, the cathode current collector (CCC) was prepared using foamed stainless steel, and the gas–liquid two-phase flow characteristics of the *μ*DMFC cathode were analyzed. Then, the foam stainless steel CCC was partially immersed in a KOH solution to complete the gradient corrosion using its capillary force. The gradient wettability CCC can create a gradient force to accelerate the drainage in the cathode of the *μ*DMFC. Compared to modified membrane electrode structures and flow field structures, the method of gradient wettability modification has the advantage of being economical, simple, and efficient. Those CCCs were classified according to the corrosion time: uncorroded CCC (N-CCC), 1 min corroded CCC (1-CCC), 5 min corroded CCC (5-CCC), and 9 min corroded CCC (9-CCC). Finally, the *μ*DMFC with different gradient wettability CCC were tested at room temperature using EIS and discharge voltage.

## 2. Methods and Experiments

### 2.1. Gas–Liquid Two-Phase Flow in the Cathode of *μ*DMFC

**Figure 1:**
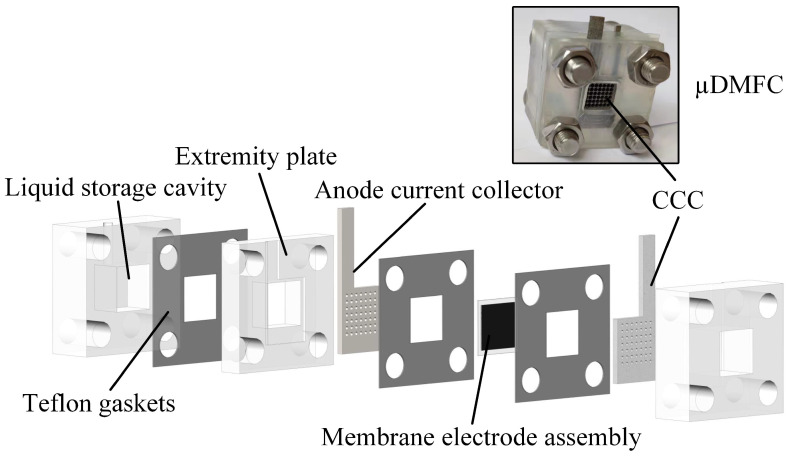
Schematic diagram of *μ*DMFC.

**Figure 2:**
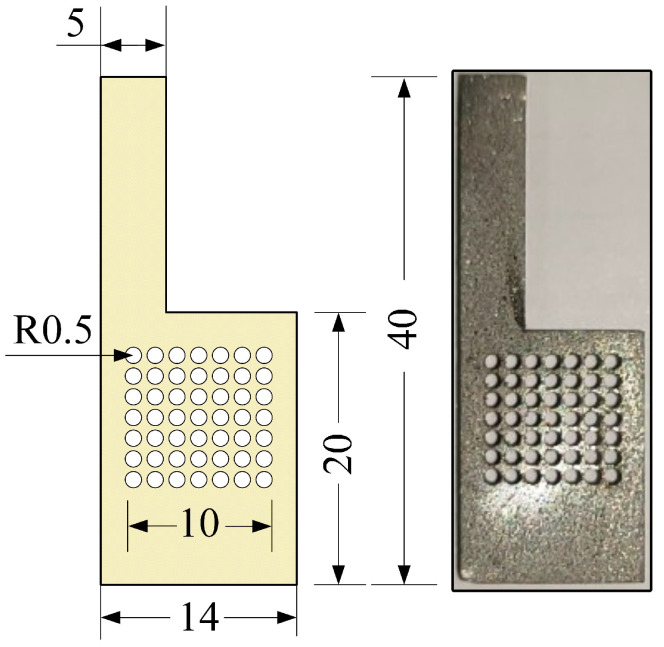
The CCC in mm.

The *μ*DMFC’s structure is shown in Figure 1. The fabrication of the various parts of the *μ*DMFC is the same as in the previous study [33]. The material of the CCC is foam stainless steel, and the flow field is of hole type, as shown in Figure 2. Then, the gas–liquid two-phase flow characteristics in the cathode of the *μ*DMFC are analyzed to find a method that can enhance the performance.

The flow state of the liquid within the CCC is related to the permeability. According to Darcy’s law:

(1) $$\Delta P_{lp}=\frac{\mu_{l}\dot{m}\Delta L}{\rho_{l}K\pi r_{eff}^{2}}$$

where $\Delta P_{lp}$ is the pressure drop of the fluid, $\dot{m}$ is the flowing mass, ΔL is the distance, $\rho_{l}$ is density, $\mu_{l}$ is kinetic viscosity, $r_{eff}$ is the effective radius of the wick, and *K* is the permeability of the CCC. The capillary pressure of the CCC is calculated as follows:

(2) $$\Delta P_{cp}=\frac{2\sigma \cos\theta }{r_{eff}}$$

where $\Delta P_{cp}$ is the capillary pressure provided by the CCC, σ is the surface tension of the H_2_O, and θ is the contact angle. When the pressure drop of the liquid flow is equal to the capillary pressure, the maximum flow rate of water in the CCC is calculated as follows:

(3) $${\dot{m}}_{l,max}=\frac{2\rho_{l}K\pi r_{eff}\sigma \cos\theta }{\mu_{l}\Delta L}$$

where ${\dot{m}}_{l,max}$ is the maximum flow rate of water in the CCC. It can be seen from Equation (3) that reducing the contact angle can increase the flow rate of water and thus the timely discharge of water that accumulates in the flow field.

Air flows mainly in a hole-type flow field, the flow state of which can be represented by the Hagen–Poiseuille equation:

(4) $$\Delta P_{ap}=\frac{32\mu_{a}{\bar{u}}_{a}\Delta L}{D^{2}}$$

where $\Delta P_{ap}$ is the pressure drop of the air, $\mu_{a}$ is the viscosity of the air, ${\bar{u}}_{a}$ is the average flow rate of the air, and *D* is the effective diameter of the hole-type flow field. Further, the oxygen flow rate is calculated as follows:

(5) $${\dot{m}}_{o}=\frac{\Delta P_{ap}\rho_{a}\pi D^{4}}{128\mu_{a}\Delta L}\times wt\%$$

where ${\dot{m}}_{o}$ is the flow rate of oxygen in the flow field, $\rho_{a}$ is the density of air, and wt% is the mass fraction of oxygen. As can be seen from Equation (5), the flow rate of oxygen is proportional to the fourth power of the effective diameter of the flow field. If the CCC is not drained in time, the hole-type flow field will be blocked by excess water, resulting in the oxygen not reaching the cathode catalytic layer smoothly.

Overall, making a wettability modification to the foam stainless steel CCC can slow down cathode flooding and thus improve the performance of the *μ*DMFC.

### 2.2. Gradient Wettability Modification for the CCC

**Figure 3:**
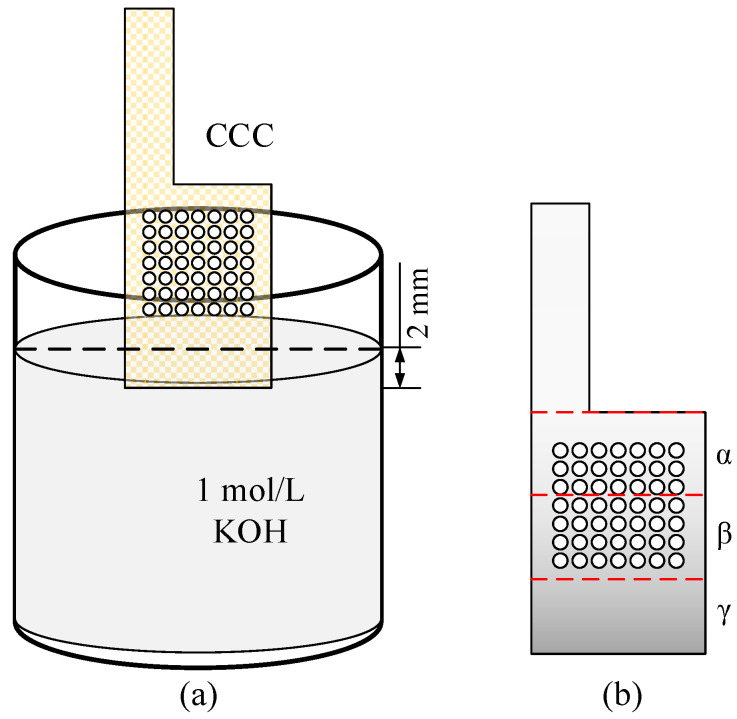
Wettability modification for the CCC. (**a**) The diagram of immersion; (**b**) Gradient partitioning.

For the gradient wettability modification of the CCC:

1. Using a laser cutting platform (Type 6060L-1000W), the CCC with the foamed stainless steel was machined, and then the surfaces of these CCC were polished smooth;
2. The CCC was cleaned with methanol, ethanol, and deionized water in turn, and then the dried CCC was immersed in the KOH solution of 1 mol/L to corrode. As shown in Figure 3a, the CCC was placed vertically with the bottom immersed to a depth of 2 mm;
3. Finally, the treated CCC was rinsed in deionized water and dried in a drying oven.

During the treatment, the bottom of the CCC was immersed in the KOH solution, and then the solution climbed upwards under the capillary force. However, due to gravitational and viscous forces, the greater the height of the CCC, the smaller the mass of the climbed solution, resulting in a weaker corrosion strength. For ease of analysis, the CCC after modification was divided equally into three regions, α, β, and γ, as shown in Figure 3b.

**Figure 4:**
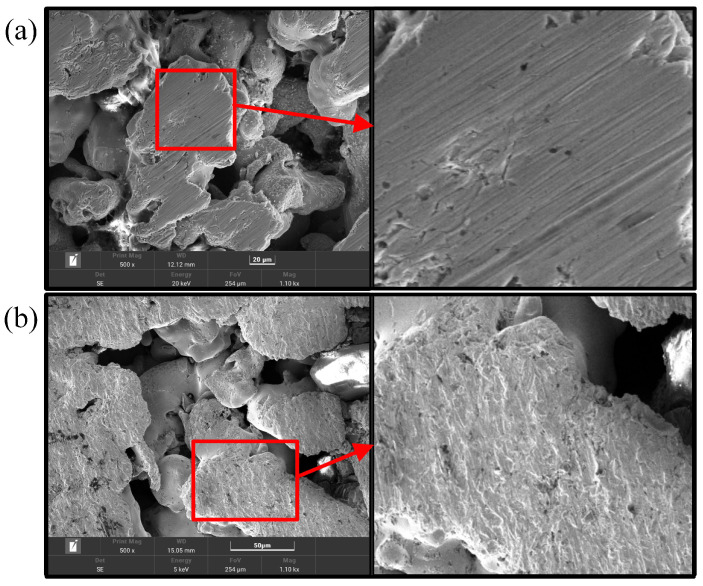
SEM of the CCC: (**a**) Before wettability modification; (**b**) After wettability modification.

The surface morphology of the CCC was analyzed. The selected equipment was TESCAN MIRA4, and an Oxford energy spectrometer was chosen. The scanning electron microscope (SEM) image shows that the surface of the CCC before the wettability modification has only a few tiny scratches, while the surface of the CCC after the wettability modification has a layer of a nanostructure, as shown in Figure 4. This layer of nanostructure corroded by the KOH solution shows a uniform character and increases the surface roughness of the CCC. Compared to the prolonged hydroxide molten salt corrosion [34] and the electrochemical corrosion of the salt solution [35], the surface morphology of the CCC after the wettability modification is not substantially damaged. Therefore, the method does not significantly destroy the CCC’s mechanical strength and electrical conductivity.

According to the Wenzel model:

(6) $$cos\theta_{w}=R_{AF}cos\theta$$

where $\theta_{w}$ is the Wenzel contact angle and $R_{AF}$ is the roughness of the wetted area. It can be seen that the nanostructure of the CCC surface can increase the surface roughness and reduce the contact angle.

The wettability modification can increase the capillary force and provide an additional capillary gradient force for the foamed stainless steel CCC. This increases the drainage capacity of the foam stainless steel CCC so that the *μ*DMFC does not flood under high-intensity operating conditions.

### 2.3. Test System for *μ*DMFC

**Figure 5:**
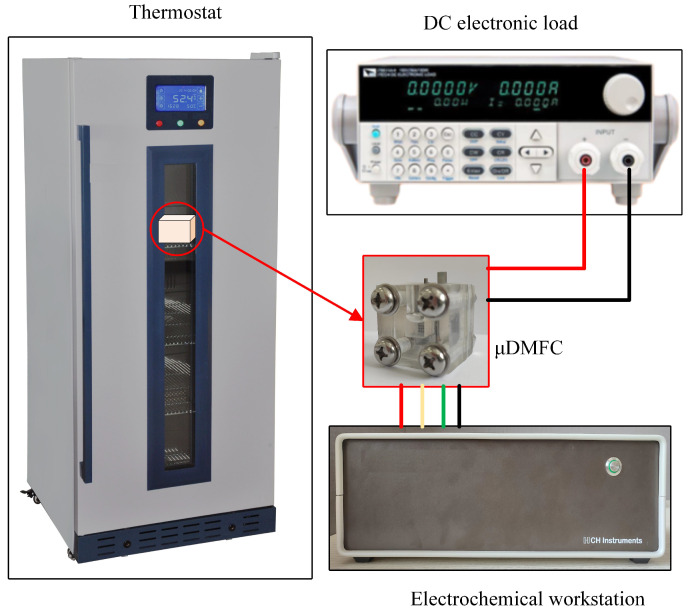
Test system of *μ*DMFC.

The test system consists of a DC electronic load, an electrochemical workstation, and a thermostat, as shown in Figure 5. This test system can perform EIS, discharge, and polarisation curve tests for the *μ*DMFC with different gradient wettability CCC. Before testing, the *μ*DMFC was activated to bring the *μ*DMFC into operation state [31,32]. Afterwards, the *μ*DMFC was placed in a 25 °C thermostat and connected to the DC electronic load and the electrochemical workstation.

## 3. Results and Discussion

### 3.1. Wettability Test

**Figure 6:**
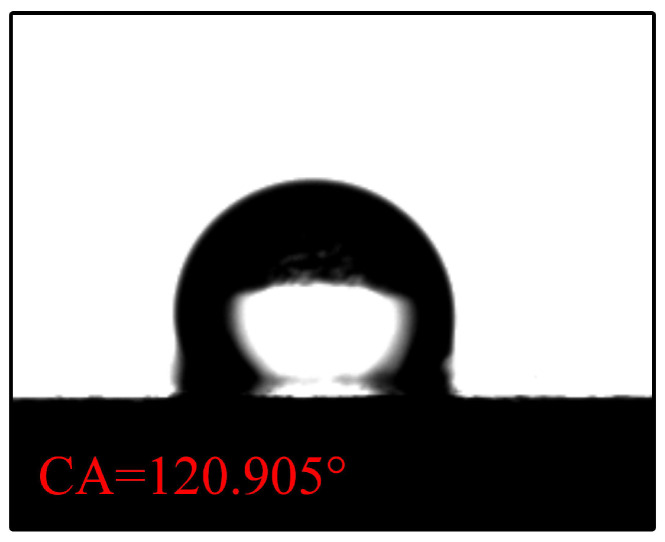
Wettability test of uncorroded CCC.

**Figure 7:**
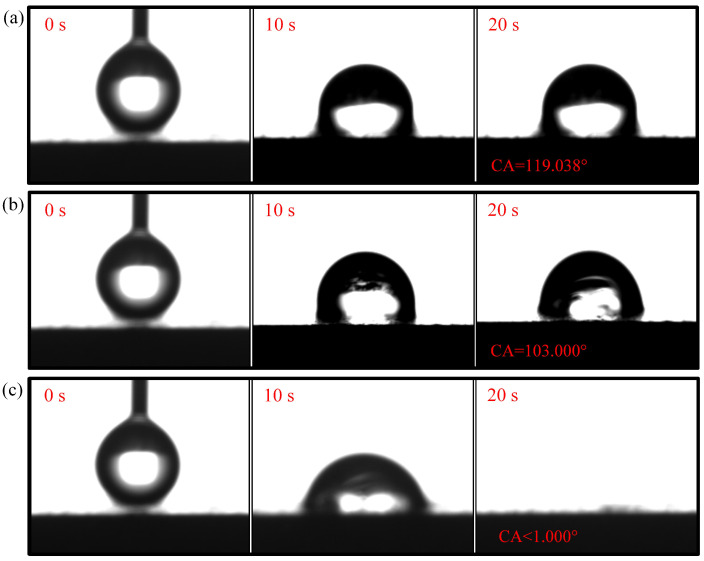
Wettability test of 1-CCC: (**a**) α region; (**b**) β region; (**c**) γ region.

**Figure 8:**
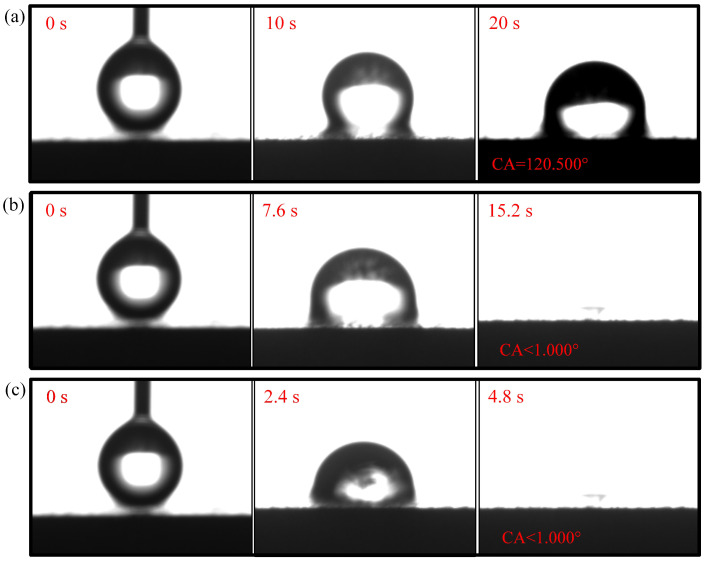
Wettability test of 5-CCC: (**a**) α region; (**b**) β region; (**c**) γ region.

**Figure 9:**
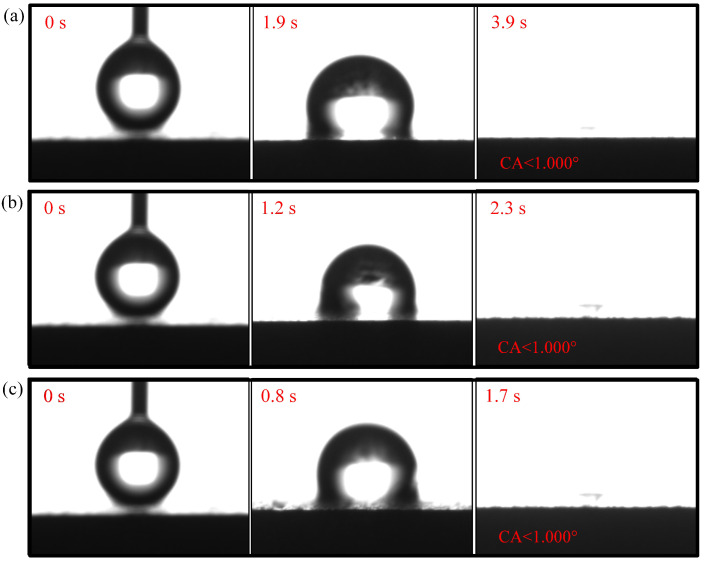
Wettability test of 9-CCC: (**a**) α region; (**b**) β region; (**c**) γ region.

The contact angle for N-CCC is $120.905^{\circ }$, as shown in Figure 6. This is mainly caused by the air inside the foamed stainless steel reducing the surface energy, which results in a hydrophobic appearance, i.e., a contact angle greater than $90^{\circ }$. After the different wettability modifications, the α, β, and γ regions of the CCC all have a hydrophilic tendency, as shown in Figure 7, Figure 8 and Figure 9.

The droplet in the α region of the 1-CCC appears in suspension, and its contact angle is always $119.038^{\circ }$. The droplet in the β region of the 1-CCC appears to complete a slow penetration, and the contact angle drops to $103.00^{\circ }$ at 20 s. In contrast, the droplet in the γ region took 20 s to penetrate completely. In the hydrophilic case, once the droplet comes into contact with the surface, it is drawn into the capillary pores by the driving force generated by the capillary effect [30,36]. From this, it is clear that the γ region of the 1-CCC is more hydrophilic than the β and α regions. Furthermore, it can be seen that the KOH solution can climb upwards along with the foamed stainless steel CCC by capillary forces to gradient-corrode the CCC. Additionally, the short immersion time results in a small mass of climbing KOH solution, and thus the β and α regions show hydrophobicity. For the 5-CCC, the droplet in the α region appears suspended, while the droplets in the β and γ regions appear to conduct a complete penetration. The penetration time of the droplets in the β and γ regions are 15.2 s and 4.8 s, respectively. This result implies that with increasing immersion time of the CCC, the mass of the climbing KOH solution increases, and thus complete permeation occurs in the β region. For 9-CCC, the α, β, and γ regions all feature complete permeation. Their permeation times are 3.9 s, 2.3 s, and 1.7 s, respectively. This implies that the longer immersion time makes the KOH solution climb upwards in large quantities, which results in severe corrosion of the entire foamed stainless steel CCC. Therefore, the overall 9-CCC displays a hydrophilic state.

The gradient behavior of the CCC after the wettability modification is shown in Table 1. According to the listed results, $119.038^{\circ }$–<1${}^{\circ }$ for 1-CCC, $120.500^{\circ }$–<1${}^{\circ }$ for 5-CCC, and $<1^{\circ }$–<1${}^{\circ }$ for 9-CCC. Furthermore, it can be seen that the hydrophilicity of the γ region of 5-CCC is superior to that of the γ region of 1-CCC because the droplet in the γ region of 5-CCC can penetrate more rapidly. Thus, the 9-CCC is almost without gradient wettability, and the 5-CCC has an optimal gradient wettability. Further, gradient wettability can generate gradient force to pull the liquid water to move directionally. The reason is that the more hydrophilic the wall, the stronger the adhesion between the droplet and the wall [3,37]. As the adhesion force increases, the contact area between the droplet and the wall increases, which causes the droplet to move towards higher hydrophilicity [38].

### 3.2. Cathode Flooding

**Figure 10:**
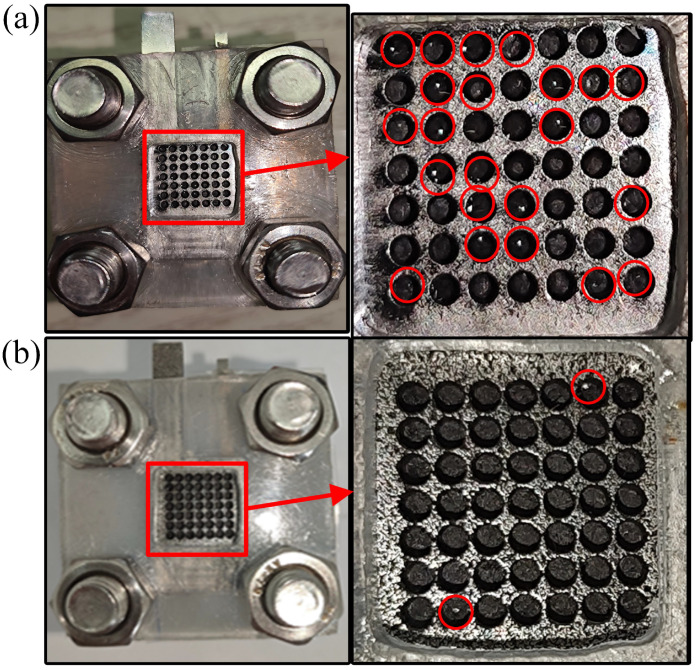
Flooding of the cathode. (**a**) *μ*DMFC of stainless steel; (**b**) *μ*DMFC of foamed stainless steel.

During the discharge process, water molecules are generated in the cathode of the *μ*DMFC. The accumulation of these water molecules for a long time will form liquid water and affect the performance of the *μ*DMFC. As shown in Figure 10, the *μ*DMFC are discharged at 50 mA/cm^2^ current to observe flooding of the hole-type flow field. It can be seen that most of the liquid water was in the cathode of the stainless steel *μ*DMFC, while little of the liquid water was in the cathode of the foamed stainless steel *μ*DMFC. This is because the liquid water of the foamed stainless steel *μ*DMFC is absorbed into the micropores by the capillary force. Moreover, this absorbing process does not change the water content inside the cathodic catalytic layer and does not affect the hydraulic pressure inside the membrane electrode assembly [30]. Therefore, the foamed stainless steel is suitable for water management studies with *μ*DMFC.

**Figure 11:**
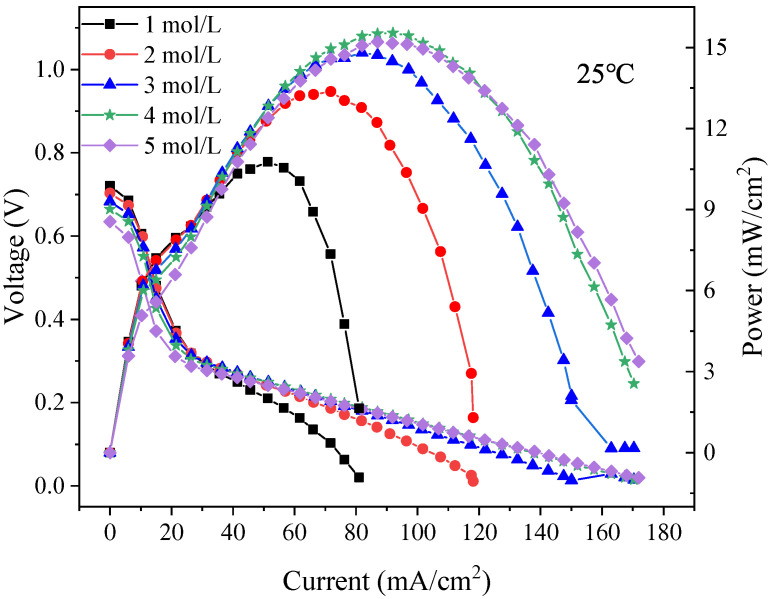
Polarization curves of foamed stainless steel *μ*DMFC at 1 mol/L–5 mol/L methanol solution concentrations.

The polarization curves of the foamed stainless steel *μ*DMFC were tested to find the optimum methanol solution concentration required for the discharge. As shown in Figure 11, the maximum power of the foam stainless steel *μ*DMFC can reach 15.5 mW at a methanol solution concentration of 4 mol/L. Therefore, the methanol solution selection is 4 mol/L. Additionally, to perform a long and stable discharge of the *μ*DMFC with different gradient wettability CCC, the current density selection is 80 mA/cm^2^, and the discharge time selection is 150 min [32].

### 3.3. EIS

**Figure 12:**
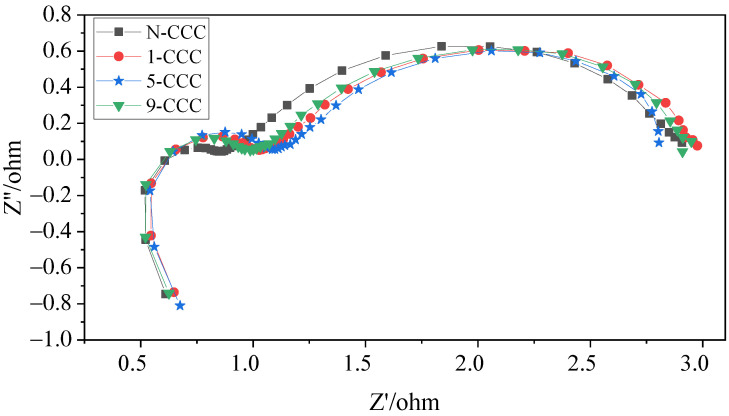
EIS of *μ*DMFC with different gradient wettability CCC before discharge.

**Figure 13:**
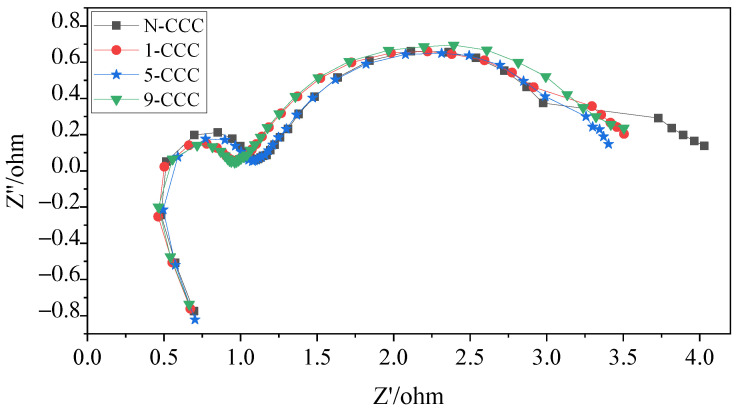
EIS of *μ*DMFC with different gradient wettability CCC after discharge.

In this work, to investigate in depth the effect of the CCC with different gradient wettability on the performance of the *μ*DMFC, AC impedance tests before and after discharge were carried out, as shown in Figure 12 and Figure 13. Their contact impedance and total impedance are shown in Table 2. It can be seen that, before discharge, the contact impedance of the *μ*DMFC with different gradient wettability CCC is 0.61Ω [39]. However, the charge transfer impedance of N-CCC *μ*DMFC is the smallest. This is because the surface of the gradient wettability CCC is corroded, which reduces its conductivity and leads to an increase in charge transfer impedance. After discharge, the EIS of the *μ*DMFC with different gradient wettability CCC changed significantly. Their total and mass transfer impedance increased significantly, but the contact impedance decreased to 0.50Ω [31,40]. This is because the water emerging in the cathode floods the catalytic layer, which increases the concentration loss and oxygen transfer resistance [1,26]. However, the water accumulated by prolonged discharge can raise the relative humidity of the reactant gas and further increase the water content in the membrane [7]. Increasing the level of membrane hydration can enhance the proton mobility and thus the electrical conductivity of the membrane [26,32].

In the low-frequency range, the *μ*DMFC of 5-CCC has the smallest curve radius, while the *μ*DMFC of N-CCC has the largest curve radius. Furthermore, the *μ*DMFC of 1-CCC and 9-CCC have essentially the same curve radius, as shown in Figure 13. Thus, the total impedance of the *μ*DMFC of 1-CCC and the *μ*DMFC of 9-CCC are essentially the same. The *μ*DMFC of 5-CCC has the lowest total impedance, while the *μ*DMFC of N-CCC has the highest total impedance. This result implies that the cathode flooding of the *μ*DMFC differs for the different gradient wettability CCC. The 5-CCC has the most suitable gradient wettability and can effectively direct water towards the end of the CCC. Hence, it can release the flow field channels and microporous channels of the CCC to provide more oxygen in the cathode of the *μ*DMFC. The 1-CCC and the 9-CCC have relatively poor capillary gradient force and cannot effectively direct water towards the end of the CCC. Thus, it cannot effectively enhance the oxygen transfer rate on the cathode side. The N-CCC does not have capillary gradient force and cannot tract water from the CCC towards the end. Thus, it has a high resistance to oxygen transfer, which leads to increased cathodic polarization.

In general, after prolonged discharge, the total impedance of the *μ*DMFC increases significantly, and the mass transfer impedance increases more than the charge transfer impedance. This implies that the effect of the mass transfer impedance on *μ*DMFC performance is much greater than that of the charge transfer impedance under prolonged discharge. The use of wettability gradient force can increase oxygen transfer channels and improve mass transfer rate. Thus, it can effectively enhance the *μ*DMFC’s performance.

### 3.4. Discharge Voltage

**Figure 14:**
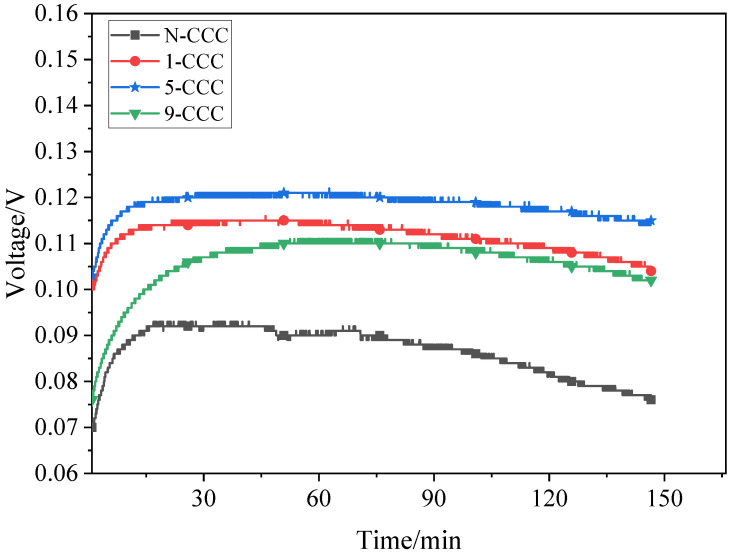
EIS of *μ*DMFC with different gradient wettability CCC after discharge.

Discharge voltage tests are carried out for different types of *μ*DMFC with a methanol solution of 2 mL, as shown in Figure 14. At 1 h, the discharge voltage of the N-CCC *μ*DMFC is 0.09 V. Compared to it, the discharge voltage of the 5-CCC *μ*DMFC, 1-CCC *μ*DMFC, and 9-CCC *μ*DMFC increased by 33.33%, 27.28%, and 23.33%, respectively. This is because the large amount of liquid water produced by the cathode during the prolonged discharge blocks the microporous channels of the CCC, making the oxygen transport path less accessible. However, the gradient wettability CCC can effectively pull the water generated in the cathode towards the end of the CCC, and the particulate water in microporous channels evaporates more quickly, which can make the microporous channels of the CCC unobstructed and increase the drainage rate and oxygen transfer rate [41,42].

During the discharge process, the performance of the N-CCC *μ*DMFC decreases the fastest while the performance of the 5-CCC *μ*DMFC decreases the slowest. Additionally, the performance of the 1-CCC and 9-CCC *μ*DMFC decreases at approximately the same rate. This implies that as the redox reaction progresses, more and more liquid water accumulates in the cathode of the *μ*DMFC. The accumulation of liquid water reduces the transfer of oxygen to the membrane electrode assembly, which increases the polarization losses and makes the local current density distribution in the *μ*DMFC very non-uniform. The *μ*DMFC with suitable gradient wettability shows better performance stability.

The performance of the *μ*DMFC is affected by the accumulation and distribution of liquid water [24]. If liquid water is not discharged from the cathode side in time, the diffusion efficiency of the gas is significantly reduced. Further, this can cause a rapid and unstable degradation in the performance of the *μ*DMFC and affect the lifetime of the *μ*DMFC [15].

## 4. Conclusions

In this study, for the foamed stainless steel *μ*DMFC, a gradient wettability CCC was prepared to avoid cathode flooding by analyzing the gas–liquid two-phase flow characteristics. Then, to find the suitable corrosion time, the wettability of four different types of CCC was tested. At room temperature, the *μ*DMFC with different gradient wettability CCC were tested using EIS and discharge voltage. The main conclusions are as follows:

1. The foamed stainless steel is more suitable to prepare the gradient wettability CCC for water management of *μ*DMFC cathode. The gradient of wettability of the 5-CCC is $120.500^{\circ }$–<$1^{\circ }$. It is significantly better than the 1-CCC and 9-CCC. At 5 min treatment time conditions, the KOH solution of 1 mol/L can provide optimal gradient corrosion for the CCC;
2. After discharge of 150 min, the 5-CCC *μ*DMFC has the lowest total impedance, whereas the N-CCC *μ*DMFC has the highest total impedance. The 5-CCC has the most suitable gradient wettability and can effectively direct water towards the end of the CCC. Thus, it has more flow field channels and microporous channels and can provide more oxygen to the cathode of the *μ*DMFC;
3. At 1 h, compared to the N-CCC *μ*DMFC, the discharge voltage of the 1-CCC *μ*DMFC, 5-CCC *μ*DMFC, and 9-CCC *μ*DMFC increased by 27.28%, 33.33%, and 23.33%, respectively. The *μ*DMFC with gradient wettability CCC shows better stability and higher discharge voltage.

## Figures and Tables

**Table 1:** Wettability of the CCC with different treatment times.

	1-CCC	5-CCC	9-CCC
α	$119.038^{\circ }$ (20 s)	$120.500^{\circ }$ (20 s)	<1${}^{\circ }$ (3.9 s)
β	$103.000^{\circ }$ (20 s)	<1${}^{\circ }$ (15.2 s)	<1${}^{\circ }$ (2.3 s)
γ	<1${}^{\circ }$ (20 s)	<1${}^{\circ }$ (4.8 s)	<1${}^{\circ }$ (1.7 s)

**Table 2:** Impedance of *μ*DMFC with different gradient wettability CCC.

	N-CCC	1-CCC	5-CCC	9-CCC
Contact impedance (before discharge)	0.61Ω	0.61Ω	0.61Ω	0.61Ω
Contact impedance (after discharge)	0.50Ω	0.50Ω	0.50Ω	0.50Ω
Total impedance (before discharge)	2.91Ω	2.95Ω	2.81Ω	2.91Ω
Total impedance (after discharge)	4.03Ω	3.51Ω	3.40Ω	3.50Ω

## Data Availability

Not applicable.

## Abbreviations

The following abbreviations are used in this manuscript:

*μ*DMFC	Micro direct methanol fuel cell
CCC	Cathode current collector
EIS	Electrochemical impedance spectroscopy
SEM	Scanning electron microscope
N-CCC	Uncorroded CCC
1-CCC	1 min corroded CCC
5-CCC	5 min corroded CCC
9-CCC	9 min corroded CCC
ΔP	Pressure drop, capillary pressure
$\dot{m}$	Flowing mass
ΔL	Distance
*ρ*	Density
*μ*	Kinetic viscosity
$r_{eff}$	Effective radius
*K*	Permeability
σ	Surface tension
θ	Contact angle
$\bar{u}$	Average flow rate
*D*	Effective diameter
wt%	Mass fraction
Subscript *l*	Liquid
Subscript *a*	Air
Subscript *o*	Oxygen
